# Editorial: Nutrition in bone health and aging

**DOI:** 10.3389/fragi.2025.1682464

**Published:** 2025-10-17

**Authors:** Joana Reis, Paulo Gavaia

**Affiliations:** ^1^ Escola Superior Agrária & CISAS, Instituto Politécnico de Viana do Castelo, Ponte de Lima, Portugal; ^2^ Center of Marine Sciences, University of Algarve, Faro, Portugal

**Keywords:** bone, osteoporosis, bone mineral density (BMD), radiomics, nutrition

Over the past few decades, research in aging-related bone diseases such as osteoporosis and osteoarthritis has made significant strides. Yet, the tangible impact of these scientific advances on the daily lives of both humans and companion animals remains limited. Progress in the area of nutrition have often been hampered and confined within disciplinary borders, resulting in a fragmented body of knowledge.

This Research Topic seeks to overcome that fragmentation by adopting a multidisciplinary perspective. Aging bone health is not merely a clinical issue, nor solely a question of molecular biology or nutrient metabolism. It is a complex confluence of factors - genetics, gender, physiological condition, co-morbidities, physical activity, environment, and habitual behaviors - that affect outcomes in a highly individualized manner.

The articles in this Research Topic collectively demonstrate the value of integrated research and point to promising paths forward. Several contributions use radiomics and imaging-based predictive models to improve early diagnosis and risk stratification for osteoporosis and related fractures - tools that can be pivotal in low-resource settings such as primary care hospitals. The contribution by Chen et al. introduce a novel screening tool for osteoporosis. By using a model based on clinical and radiomic features, this toll has the potential for democratizing osteoporosis screening in underserved regions. Yu et al. present a new lumbar CT-based radiomics model for predicting osteoporotic vertebral compression fractures (OVCF) in postmenopausal women, offering a possible alternative to traditional bone mineral density (BMD) assessments. Notably, this model outperformed DEXA scans in sensitivity and accuracy, even among patients with similar T-scores, highlighting the inadequacy of relying on single metrics in isolation and reinforcing the need for individualized risk assessment.

Beyond imaging, biochemical and nutritional biomarkers should also be further explored and integrated into the risk assessment and diagnosis processes. The study by Liu et al. shows as albumin-corrected anion gap (ACAG) levels correlate with reduced lumbar spine BMD, suggesting a novel metabolic marker for osteoporosis risk - though the relationship is nonlinear.

The study by Li et al. further deepens the nutritional lens on aging and bone health: the BMD of the lumbar spine was positively correlated with serum 25-hydroxyvitamin D levels, the difference between actual body weight and ideal body weight, and uric acid levels in the blood. Conversely, the BMD of the femoral neck was negatively correlated with age and female sex, and positively correlated with height and geriatric nutrition risk index score, suggesting a protective role of uric acid over bone in advanced age, and underscoring the necessity for personalized dietary recommendations.

The article by Guo et al. found sex- and bone-site-specific variations in the associations between vitamin K intake levels and bone health in individuals aged over 50 years.

The intersection of bone health with cardiovascular and muscular health adds further layers to the picture.

The study by Westbury et al. illustrates how calcium supplementation - commonly recommended for bone health - may paradoxically increase fracture risk, possibly due to reverse causality, while dietary calcium shows protective effects against cardiovascular mortality. Whilst dietary calcium intake was not associated with either overall fracture or hip fracture, a better-quality diet was related to reduced risk of hip fracture, after adjustment for sex. Such findings call for more nuanced dietary guidelines that go beyond isolated nutrients and consider long-term patterns and holistic outcomes. Importantly, prudent diet scores were also associated with beneficial lifestyle traits, such as higher physical activity and lower rates of smoking, reflecting the broader complexity of the socio-behavioral ecosystem in which nutrition operates. The systematic review and metanalysis by Yan et al. identified osteoporosis as a significant risk factor for development of stroke related sarcopenia, along with age, tube feeding, pre-stroke sarcopenia, atrial fibrillation, neurological compromise (as evaluated by the NIHSS score).

Finally, the study by Liu et al. explores the relationship between the use ofdiuretics and fracture risk. Diuretics are used widely in the treatment of conditions such as hypertension, cardiac and kidney disease. This study specifically approaches the differing effects of loop, thiazide, and potassium-sparing diuretics, reinforcing the critical need for integrated pharmacological and nutritional strategies, and further research, especially in aging populations vulnerable to falls and bone injuries.

Taken together, the articles in this Research Topic demonstrate that advancing bone health in aging requires a system-level approach, grounded in real-world variability. We encourage clinicians, researchers, veterinarians, nutritionists, and public health professionals to embrace a framework that bridges disciplines and actively translates findings into interventions tailored to individual contexts.

The editors would like to thank the excellent contributions of the reviewers and editorial team to the Research Topic here presented.

We hope that this Research Topic will provide meaningful insights to the development of practical, evidence-based tools and strategies that are responsive to complexity, rather than reductive. Only through such an approach can we move from fragmented insights to holistic solutions—benefiting the health and longevity of the living beings we care for.

